# Application of ionic liquid ultrasound-assisted extraction (IL-UAE) of lycopene from guava (*Psidium guajava* L.) by response surface methodology and artificial neural network-genetic algorithm

**DOI:** 10.1016/j.ultsonch.2024.106877

**Published:** 2024-04-16

**Authors:** Junping Wang, Hongyi Zhao, Xuexue Xue, Yutong Han, Xin Wang, Zunlai Sheng

**Affiliations:** a College of Veterinary Medicine, Northeast Agricultural University, Harbin 150030, PR China; b Heilongjiang Key Laboratory for Animal Disease Control and Pharmaceutical Development, Harbin 150030, PR China

**Keywords:** Lycopene, *Psidium guajava* L., Ionic liquids, Ultrasound-assisted extraction (UAE), Artificial neural network-genetic algorithm (ANN-GA), Response surface methodology (RSM)

## Abstract

Lycopene-rich guava (*Psidium guajava* L.) exhibits significant economic potential as a functional food ingredient, making it highly valuable for the pharmaceutical and agro-food industries. However, there is a need to enhance the extraction methods of lycopene to fully exploit its beneficial uses. In this study, we evaluated various ionic liquids to identify the most effective one for extracting lycopene from guava. Among thirteen ionic liquids with varying carbon chains or anions, 1-butyl-3-methylimidazolium chloride demonstrated the highest productivity. Subsequently, a single-factor experiment was employed to test the impact of several parameters on the efficiency of lycopene extraction using this selected ionic liquid. These parameters included extraction time, ultrasonic power, liquid-solid ratio, concentration of the ionic liquid, as well as material particle size. Moreover, models of artificial neural networks using genetic algorithms (ANN-GA) and response surface methodology (RSM) were employed to comprehensively assess the first four key parameters. The optimized conditions for ionic liquid ultrasound-assisted extraction (IL-UAE) were determined as follows: 33 min of extraction time, 225 W of ultrasonic power, 22 mL/g of liquid-solid ratio, 3.0 mol/L of IL concentration, and extraction cycles of three. Under these conditions, lycopene production reached an impressive yield of 9.35 ± 0.36 mg/g while offering advantages such as high efficiency, time savings, preservation benefits, and most importantly environmental friendliness.

## Highlights

• Ionic liquids were efficient in extracting lycopene from guava.
• [C_4_mim]^+^Cl^−^ increased the extraction compared to organic solvents.
• Extraction of lycopene from guava was modeled using (RSM) and (ANN).
• ANN-GA showed better precision than RSM for the UAE method of lycopene from guava.

## 1 Introduction

Guava (*Psidium guajava* L.) is an important crop belonging to the genus Psidium and the Myrtaceae family, originating in the tropics of South America. It was brought to many parts of the world and is now naturalized there [1]. Guava is abundant in V_C_, soluble sugar, soluble protein, and various polyphenolic substances [2]. Owing to its substantial medicinal and nutritional value, it enjoys great popularity among consumers. Lycopene (C_40_H_56_) is a natural edible colorant belonging to the carotene group, widely found in tomatoes, guava, watermelons, and other fruits [3]. Lycopene exhibits diverse biological functions including antioxidant activity, anti-cancer properties, nucleic acid damage reduction, cardiovascular disease prevention, genetic mutation suppression, blood lipid reduction as well as immune regulation [4], [5], [6]. It has been reported that lycopene possesses more than twice the antioxidant capacity of beta-carotene, which is one of the strongest antioxidants in nature that delay aging [7]. Furthermore, lycopene can be incorporated into cosmetic formulations to develop products that effectively slow down skin aging [8]. With the continuous in-depth study of lycopene, scientific evidence has demonstrated the effective inhibition of esophageal cancer, prostate cancer, leukemia, etc., by lycopene [9], [10], [11].

Lycopene is an important source of antioxidant activity in some plants. Since the degradation of this compound in the heating process affects its antioxidative activity [12], UAE emerges as an efficient method for extracting lycopene from guava due to ultrasonic cavitation-induced shear forces that mechanically disrupt cell walls and facilitate material transfer, thereby minimizing potential chemical degradation of target compounds [13]. Furthermore, UAE exhibits a greater recovery of bioactive components and calls for a shorter extraction time, a lower extraction temperature, and less organic solvent than conventional extraction techniques including maceration, Soxhlet, and percolation [14], [15]. However, the extraction duration, liquid–solid ratio, and ultrasonic power all play a part in the intricate process of UAE. The yields of bioactive compounds can be impacted by these parameters separately or in combination. Additionally, cutting-edge multivariate analytic techniques like the artificial neural network-genetic algorithm (ANN-GA) and response surface methodology (RSM) may be applied to improve extraction settings and validate the independent and combined impacts of these variables [16], [17]. By creating a quadratic equation, RSM, a statistical method frequently used in analytical optimization, may investigate the impact of several factors on the goal answer. As ANN-GA is a relatively new and well-liked non-linear computational modeling technique, it has been utilized as a supplemental method for the optimization of effective constituents. However, there hasn't been a publication on a comparison between ANN-GA and RSM for lycopene extraction optimization from guava so far.

Some traditional extraction solvents, such as ethanol, petroleum mercury, chlorophyll, and methamphetamine, have a high cost and serious disadvantages to environmental pollution. Due to their low toxicity and good solubility, ILs are often referred to as environmentally friendly alternatives, have garnered considerable attention across various fields. ILs have exhibited promising solvent capabilities for extracting diverse beneficial chemicals from plant samples, including terpene essential oil and procyanidins [18], alkaloids [19], polyphenolic compounds [20], and phenolic acid [21]. Therefore, it is worthwhile to study lycopene's ultrasonic extraction utilizing IL aqueous solution.

This work aims to create an efficient, quick, and eco-friendly process for extracting lycopene from guava using IL-UAE. Using artificial neural network-genetic algorithm (ANN-GA) and response surface methodology (RSM) techniques, this study carefully examines many ILs with different cations and anions in order to optimize ultrasonic power, IL concentration, liquid–solid ratio, and extraction time.

## 2 Materials and method

### 2.1 Materials and apparatus

The fresh fruits of guava were obtained from Liuzhou City, Guangxi Province, China in September 2023. Associate Prof. Xueying Chen of the Northeast Agricultural University authenticated the guava. Subsequently, the fruits was naturally dried at ambient temperature, crushed, and stored at 4 ^◦^C until further use. Lycopene with a purity of 98 % came from the Chinese Institute for the Control of Pharmaceutical and Biological Products (Beijing, China, Lot: L465063). All ILs used in this study were obtained from Aladdin Reagent Co., Ltd (Shanghai, China) and utilized as received without any modifications. Other analytical grade reagents were procured from Kemiou Chemical Reagent Co., Ltd (Tianjin, China).

A KQ-250DB ultrasonic unit (Kunshan, Jiangsu, China) with a maximum power of 250 W was utilized in this study. The apparatus consisted of a rectangular container measuring 23.5 cm by 13.3 cm by 10.2 cm, equipped with annealed transducers operating at a frequency of 50 kHz and attached to the base. Lycopene analysis of the sample was performed using a UV-6000 UV–Vis spectrophotometer (Shanghai, China).

### 2.2 Determination of lycopene content

The lycopene content was determined through the colorimetric approach [22] with certain modifications. Initially, 1 mg of lycopene with a purity level of 98 % was measured and dissolved in petrol ether solution. Subsequently, after being moved to a 50 mL volumetric flask, the solution was diluted to volume using petrol ether as the stock solution. Next, different 10 mL volumetric flasks were filled with varying volumes (1–6 mL) of the stock solution, which was then further diluted to volume with petrol ether. The absorbance values were measured at an optical wavelength of 502 nm against petrol ether as blank using UV-6000 spectrophotometer. The calibration curve obtained for this experiment ranged from concentrations of lycopene between 2 μg/mL to 12 μg/mL (R^2^ = 0.9992), following the equation y = 0.064x + 0.0279, where y represents absorbance value and x denotes sample concentration.

### 2.3 The screening of ILs

To compare different ILs on their extraction efficiency in this study, nine types of anions including OAC^−^, Cl^−^, HSO_4_^−^, BF_4_^−^, Br^−^, HPF_6_^−^, NO_3_^−^, FeCl_4_^−^, and TFS^−^ as well as five cations featuring various carbon chain lengths ([C_2_mim]^+^, [Amim]^+^, [C_6_mim]^+^, [Bzmim]^+^ and [C_4_mim]^+^) were employed. In each conical flask, a combination consisting of dried material weighing 1.0 g and aqueous solutions containing ionic liquid at a concentration of 0.5 mol/L was prepared. The resulting mixture was subjected to ultrasound treatment within an ultrasound bath, and the following additional extraction parameters were employed: ultrasonic power of 250 W, liquid-solid ratio of 20 mL/g, and extraction time of 30 min. Subsequently, the reaction mixture was collected and centrifuged for 5 min at 3000 rpm. The resulting supernatant was aspirated (1 mL) and diluted with methanol to achieve the desired concentration. The absorbance values of these sample solutions were measured at 502 nm against the same mixture, without the sample as a blank. Then the absorbance was calculated as the concentration of lycopene from the calibration curve in section 2.2.

### 2.4 Single-factor screening

In a conical flask, a combination of 1.0 g dried material and an aqueous solution containing the IL was prepared. The conical flask was partially submerged in an ultrasonic bath thereafter. Optimization experiments were conducted by varying IL concentration (ranging from 0.5 to 3.5 mol/L), liquid-solid ratio (ranging from 10 to 30 mL/g), extraction time (ranging from 10 to 50 min), material particle size (ranging from 20 to 120 mesh), and ultrasonic power (ranging from 100 to 250 W).

### 2.5 Optimization UAE methods by RSM

Based on the single-factor experiments results, the Box-Behnken design (BBD) methodology was employed for further optimization of four continuous variables: IL concentration, extraction time, liquid–solid ratio, and ultrasonic power settings (Table 1). Predicted optimal values along with their corresponding experimental conditions were screened to determine the most favorable extraction conditions. The experiment involved a four-factor three-level test design employing 29 random combinations fitted into a second-order polynomial model. A fitting equation is provided below:

(1) $$Y=\beta_{0}+\sum_{i=1}^{k}\beta_{i}x_{i}+\sum_{i=1}^{k}\beta_{\mathrm{ii}}X_{i}^{2}+\sum_{i=1}^{k-1}\sum_{j=i+1}^{k}\beta_{\mathrm{ij}}X_{i}X_{j}$$

The dependent variable, denoted as Y in this equation, is influenced by the independent variables X_i_ and X_j_. The coefficients β_0_, β_i_, β_ii_, and β_ij_ represent the intercept, linear, quadratic, and interaction effects, respectively. The number of independent parameters k is 4 in this case.

**Table 1:** Coding of experimental parameters and related levels.

Experimental	Unit	Symbols	Coded values		
parameters		(X_i_)	Low (−1)	Medium (0)	High (+1)
Extraction time	min	X_1_	20	30	40
Ionic liquid concentration	mol/L	X_2_	2.5	3	3.5
Liquid-solid ratio	mL/g	X_3_	15	20	25
Ultrasonic power	W	X_4_	150	200	250

### 2.6 Optimization UAE methods by artificial neural networks-genetic algorithm

To train the artificial neural network (ANN) using MATLAB R2019b software (MathWorks, Natick, USA), experimental data values of the RSM simulation were utilized. A three-layer feed-forward error back propagation ANN was modeled using the Sheffield neural network toolbox. It consists of an input layer with four neurons, a hidden layer with nine neurons, and an output layer with one neuron. By normalizing the distribution data, it can enhance the correlation coefficient between the independent and dependent parameters. To achieve minimum RMSE values, the input and target data of individual ANN nodes were normalized within a range of 0 (new x_min_) to 1 (new x_max_), facilitating rapid convergence.

The ANN model was optimized using a genetic algorithm with a maximum of 100 iterations, a population size of 40, and a crossover probability of 0.75 %. After evaluating individuals, genetic processes including reproduction, cross-over, and mutation were performed to generate the next generation. The fitness function was used to select the best chromosomes for reproduction which were then passed on to subsequent generations. During cross-over, distinct components from two individuals were combined to create offspring while chromosomes undergo random alterations during mutation. This process continued until an almost ideal solution was found or termination requirements were met.

### 2.7 Effect of extraction cycles on extraction efficiency

The fruits in the aforementioned experiments were extracted only once; however, it is important to study the extraction cycles of the sample to enhance the efficiency of the extraction process. Therefore, this study examined the impact of various extraction cycles (numbers 1, 2, 3, 4, 5, and 6) on the yield of extraction. The six sample groups were obtained using the optimal parameters identified earlier.

### 2.8 Comparison with conventional methods

Pure water was selected as a reference solvent for ultrasound-assisted extraction (UAE) of lycopene from guava. Apart from the solvent type, all other aspects of the extraction experiment were conducted under optimal conditions. Specifically, a dried plant sample weighing 1 g was mixed with 20 mL of pure water and subjected to UAE for 30 min at an ultrasonic power level of 250 W. Subsequently, the extract was filtered and diluted with water to achieve the desired concentration. The absorbance values of the solution was measured at 502 nm against the solvent as a blank. Then the absorbance was calculated as concentration of lycopene from the calibration curve in section 2.2.

For heat reflux extraction (HRE), 80 % ethanol solution was selected as the solvent. A dried plant sample weighing 1 g was refluxed with 20 mL of 80 % ethanol solution for a period lasting two hours. Subsequently, the extract was filtered and diluted with the solvent. The absorbance value of the sample solution was measured at 502 nm against 80 % ethanol solution as a blank. Then the absorbance was calculated as concentration of lycopene from the calibration curve in section 2.2.

### 2.9 Scanning electronic microscopy (SEM) analysis

The surface morphology and form of three different guava powder samples were further investigated utilizing scanning electron microscopy (SU8010, Hitachi Ltd., Japan) operating in a high vacuum at an acceleration voltage of 18 kV. The three samples were as follows: A) air-dried sample collected after UAE treatment with pure water; B) air-dried sample collected after heat reflux extraction with aqueous ethanol (80 %); C) air-dried sample collected after UAE treatment with IL solution. Measurements were taken at magnification levels ranging from 150 × to 1000 × for multiple samples.

### 2.10 Statistical analysis

Design Expert 13.0 (Stat-Ease Inc., Minneapolis, MN, USA) and MATLAB R2019b (MathWorks, Natick, MA, USA) were the programs used to statistically analyze the experimental data. In each experiment, the results are shown as mean ± standard deviation (SD) from at least three different studies (n = 3). Sample comparisons were evaluated using one-way analysis of variance (ANOVA) and *F*-test, considering differences significant at *p* < 0.05 level. Design Expert 13 was utilized for statistical design and optimization based on response surface methodology (RSM). This led to regression equations for assessed responses, determination of parameter contribution and importance, generation of response surface plots, as well as identification of optimal and sub-optimal extraction strategies. MATLAB R2019b was employed for building, testing, and validating ANN-GA.

## 3 Results and discussion

### 3.1 Effects of ILs on the lycopene yield

#### 3.1.1 Screening of the anions

**Fig. 1:**
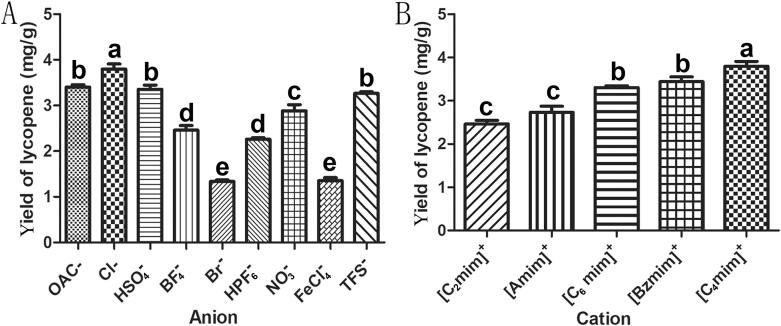
Selection of ionic liquid solvent system in the model of ultrasound-assisted extraction of lycopene from guava. (A) The type of anions, (B) imidazole cations with different alkyl chain lengths. Data are means ± SD, n = 3 for each bar. Different low case letters above columns indicate statistical differences at* p* < 0.05, same letters indicate insignificantly different (*p* > 0.05).

To investigate the impact of different anions on the lycopene yield, this study examined nine distinct anions ([BF_4_]^−^, [HPF_6_]^−^, Br^−^, [NO_3_]^−^, [OAc]^−^, [TFS]^−^, Cl^−^, [FeCl_4_]^−^, [HSO_4_]^−^) present in 1-butyl-3-methylimidazole. Fig. 1A demonstrates that the extraction yield of lycopene is significantly influenced by various types of anions in ILs. Notably, Cl^−^ exhibited the highest average yield of lycopene extraction at 3.80 ± 0.11 mg/g compared to other anions. Conversely, Br^−^ displayed a poor value with an average yield of lycopene extraction at only 1.34 ± 0.03 mg/g. These findings suggest that incorporating Cl^−^ as an anion overcomes the challenge posed by low water solubility and enhances lycopene extraction efficiency.

#### 3.1.2 Screening of cation alkyl chain length

The anion screening results indicated that Cl^−^ was the optimal choice for lycopene production. To investigate the impact of different cation alkyls on lycopene extraction, four cations ([C_4_mim]^+^, [C_2_mim]^+^, [Amim]^+^, [C_6_mim]^+^ and [Bzmim]^+^) were tested. As depicted in Fig. 1B, the lycopene yield increased with the elongation of alkyl chain length. Notably, C_4_ imidazole exhibited the highest extraction yield of lycopene (3.80 ± 0.11 mg/g). However, a continuous improvement in extraction yield was not observed as the alkyl chain lengthened; instead, a slight reduction was noticed. Moreover, the mean yields of lycopene in the presence of [C_6_mim]^+^ and [Bzmim]^+^ were 3.31 ± 0.12 mg/g and 3.44 ± 0.11 mg/g, respectively.

This phenomenon can be credited with the increasing lipophilicity of ionic liquids with longer alkyl chains in their cations due to a similar miscibility principle. Nevertheless, as the alkyl chain lengthens further in cations, steric hindrance becomes more significant than lipophilicity resulting in decreased lycopene yield. This behavior is also evident in ILNAS where a correlation between imidazole-based IL's cationic alkyl chain length and maximum extraction yield is observed for five alkaloids [19].

### 3.2 Parameter optimization using single-factor design

#### 3.2.1 Effect of extraction time on the lycopene yield

**Fig. 2:**
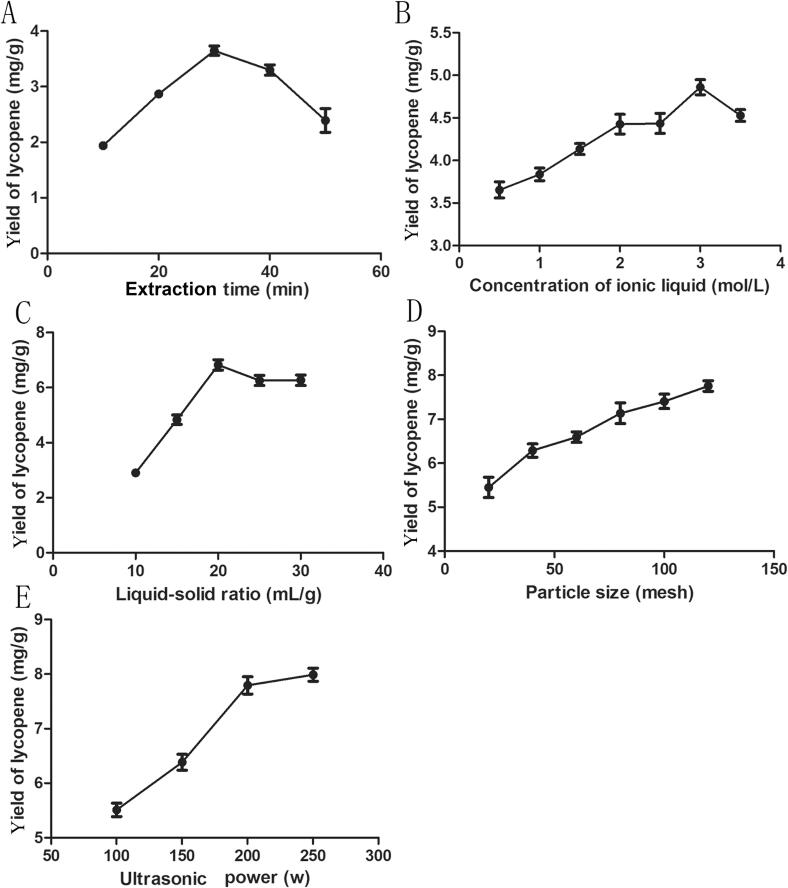
Effects of different factors on lycopene yield from guava. (A) extraction time, (B) concentration of ionic liquid, (C) liquid–solid ratio, (D) particle size, and (E) ultrasonic power.

One important consideration in reaching dissolution equilibrium is the extraction time. As a result, the effects of various extraction times (10–50 min) were tested. Fig. 2A showed that as extraction time increased, the lycopene yield initially rose and subsequently declined. After 30 min, the maximum yield of lycopene (3.80 ± 0.11 mg/g) was produced. These results indicate that extending the extraction time beyond 30 min does not significantly affect lycopene extraction efficiency; thus, an ultrasonic irradiation duration between 20 to 40 min was determined as optimal in this experiment.

It is widely recognized that the content of active substances in biomass remains constant and there is a limited capacity for extracting these substances into the solvent during the process [23]. Consequently, typically observed behavior involves an initial increase followed by stabilization in active substance yield over time. However, contrary to this general trend, our study found that as the duration increased, lycopene yield initially rose but subsequently declined due to both dissolution equilibrium considerations and instability issues within the IL solution.

#### 3.2.2 Effect of IL concentration on the lycopene yield

To find the ideal IL concentration, lycopene yield was assessed at different IL concentrations (0.5 mol/L-3.5 mol/L). The lycopene yield rose initially and subsequently somewhat declined as the IL concentration rose, reaching a peak value of 4.86 ± 0.12 mg/g at 3.0 mol/L (Fig. 2B). Consequently, an ideal concentration of 3.0 mol/L [C_4_min] [Cl] solution was determined.

This phenomenon can be attributed to the similar miscibility principle that causes ionic liquids to become more lipophilic as IL concentration rises. Unfortunately, the solvent becomes extremely viscous due to the high concentration of ionic liquid, which makes it impossible for the solvent to pass through plant tissue and inappropriate for extracting the active ingredients from plants [24]. This was in line with what we found in Fig. 2B, where the lycopene production trended lower at 3.5 mol/L ionic liquid concentrations.

#### 3.2.3 Effect of liquid–solid ratio on the lycopene yield

The extraction process is still significantly influenced by the liquid–solid ratio. Insufficient solvent quantities may lead to incomplete extraction while excessive solvent generates unnecessary waste products. Fig. 2C illustrated the effects of various liquid–solid ratios (10–30 mL/g) on the lycopene yield. Within the range of 10 to 20 mL/g for the liquid–solid ratio, the lycopene yield rose quickly and got a maximum value (6.82 ± 0.17 mg/g) at 20 mL/g. Later, the lycopene yield somewhat decreased as the ratio grew. The optimal liquid–solid ratio for lycopene extraction was found to be 20 mL/g in order to reduce solvent waste. In the current optimization investigation, the liquid–solid ratios of 15–25 mL/g were therefore employed.

#### 3.2.4 Effect of particle size on the lycopene yield

In general, particle size reduction is a pretreatment technique that enhances the efficient extraction of active compounds from plants. Therefore, this study investigated the influence of different particle sizes (20–120 mesh) on lycopene yield. As depicted in Fig. 2D, the yields of lycopene exhibited an initial increase followed by a plateau as the particle size decreased from 20 to 120 mesh. Notably, at 100 or 120 mesh, the yield of lycopene reached its peak value (7.78 ± 0.17 mg/g).

The underlying rationale behind this observation is that reducing the material's particle size exposes a greater certain surface area to the extraction solvent, thereby enhancing mass transfer efficiency. Consequently, considering green chemistry principles, we selected a mild degree of crushing (100 mesh) as our ideal extraction condition to minimize energy loss and and pollutants to the environment that comes with overcrushing.

#### 3.2.5 Effect of ultrasonic power on the lycopene yield

The impact of the ultrasonic power (100–250 W) variable on the lycopene yield was investigated since this variable is crucial for ensuring effective extraction. As shown in Fig. 2E, the lycopene yield consistently rose with increasing ultrasonic power, and peaked (8.49 ± 0.13 mg/g) at 250 W. This implies that increasing the power of the ultrasonic irradiation raises the bubble's internal pressure and temperature, which enhances the extractant's cavitation, speeds up the solvent's entrance into the cytoplasmic matrix, and encourages the extraction of the sample. Thus, the 150–250 W of ultrasonic power was further improved by RSM.

### 3.3 RSM optimization

#### 3.3.1 The model fitting and statistical analysis

To maximize the extraction of lycopene from guava, the RSM approach was utilized. A total of 29 planned tests were conducted to optimize each unique parameter in the Box-Behnken design. The experimental setup and lycopene production based on the factorial design are presented in Table 2. Based on the application of multiple regression analysis to the experimental data, the response variable and the test variables were related by the subsequent second-order polynomial equation:

(2) Y = −89.51275 + 0.8045*X_1_* + 29.41733*X_2_* + 2.50752*X_3_* + 0.137552*X_4_* + 0.0247*X_1_X_2_* + 0.001005*X_1_X_3_* + 0.000632*X_1_X_4_* − 0.0021*X_2_X_3_* − 0.00193*X_2_X_4_* + 0.001484*X_3_X_4_* − 0.016896*X_1_^2^* − 5.02633*X_2_^2^* − 0.065163*X_3_^2^* − 0.000426*X_4_^2^*

**Table 2:** List of experimental values and predicted values from RSM and ANN.

Run	Independent variables				The yield of lycopene (mg/g)		
	Extraction time (min)	Ionic liquid concentration (mol/L)	Liquid-solid ratio (mL/g)	Ultrasonic power (W)	Actual values	RSM	ANN
						predicted	predicted
1	20	2.5	20	200	5.57	5.54	5.58
2	40	2.5	20	200	5.85	5.52	5.75
3	20	3.5	20	200	4.9	4.87	4.82
4	40	3.5	20	200	5.67	5.34	5.7
5	30	3	15	150	4.53	4.33	4.35
6	30	3	25	150	6.09	5.81	6.24
7	30	3	15	250	4.67	4.6	4.66
8	30	3	25	250	7.71	7.56	7.65
9	20	3	20	150	5.32	5.21	5.06
10	40	3	20	150	4.85	4.81	4.45
11	20	3	20	250	5.42	5.59	5.4
12	40	3	20	250	6.22	6.45	6.31
13	30	2.5	15	200	4	4.48	3.98
14	30	3.5	15	200	4.3	4.06	4.18
15	30	2.5	25	200	6.36	6.71	6.63
16	30	3.5	25	200	6.64	6.27	6.63
17	20	3	15	200	3.88	3.77	3.7
18	40	3	15	200	3.78	3.9	3.54
19	20	3	25	200	5.78	5.89	5.88
20	40	3	25	200	5.88	6.22	6.03
21	30	2.5	20	150	5.64	5.61	5.54
22	30	3.5	20	150	4.6	5.28	4.65
23	30	2.5	20	250	7.15	6.71	7.04
24	30	3.5	20	250	5.91	6.19	5.86
25	30	3	20	200	8.41	8.27	8.22
26	30	3	20	200	8.4	8.27	8.22
27	30	3	20	200	8.13	8.27	8.22
28	30	3	20	200	8.02	8.27	8.22
29	30	3	20	200	8.38	8.27	8.22

where the coded values for extraction time, IL concentration, liquid–solid ratio, and ultrasonic power were represented by the letters *X_1_*, *X_2_, X_3_*, and *X_4_*, respectively.

The *F*-test was utilized to determine the significance of the quadratic model and to evaluate the statistical significance of the regression equation. Table 3 provides an overview of the ANOVA for the response surface quadratic polynomial model. Each coefficient's significance as well as the intensity of each parameter's interaction were determined using the *p*-value. The importance of the associated coefficient increased with decreasing *p*-values. In this case, the model's *p*-value was less than 0.0001, indicating that it was appropriate for use in this experiment. The Predicted R^2^ of 0.8038 was in reasonable agreement with the Adjusted R^2^ of 0.9286; i.e. the difference was less than 0.2, which indicated a good agreement between the projected and real values. The lack-of-fit term for the model (*p* > 0.05) indicates that the experiment has a modest error and good fit, the model residuals are generated by random errors, and the differences are not statistically significant. As can be observed from the *F* and *p* figures, the liquid–solid ratio, ultrasonic power, IL concentration, and extraction time, in that order, are the parameters influencing the lycopene yield.

**Table 3:** ANOVA for Quadratic model.

Source	Sum of Squares	df	Mean Square	F-value	**p-value**	
**Model**	54.58	14	3.9	27	< 0.0001	Significant
X_1_-ultrasonic time	0.1573	1	0.1573	1.09	0.3143	
X_2_-IL concentration	0.5487	1	0.5487	3.8	0.0716	
X_3_-liquid–solid ratio	14.74	1	14.74	102.05	< 0.0001	
X_4_-ultrasonic power	3.05	1	3.05	21.12	0.0004	
X_1_X_2_	0.061	1	0.061	0.4225	0.5262	
X_1_X_3_	0.0101	1	0.0101	0.0699	0.7953	
X_1_X_4_	0.4001	1	0.4001	2.77	0.1182	
X_2_X_3_	0.0001	1	0.0001	0.0008	0.9783	
X_2_X_4_	0.0093	1	0.0093	0.0645	0.8032	
X_3_X_4_	0.5506	1	0.5506	3.81	0.0712	
X_1_2	18.52	1	18.52	128.23	< 0.0001	
X_2_2	10.24	1	10.24	70.93	< 0.0001	
X_3_2	17.21	1	17.21	119.21	< 0.0001	
X_4_2	7.35	1	7.35	50.91	< 0.0001	
**Residual**	2.02	14	0.1444			
Lack of Fit	1.89	10	0.1893	5.89	0.0511	Not significant
Pure Error	0.1286	4	0.0321			
**Cor Total**	56.6	28				

#### 3.3.2 Evaluation of the response surface

**Fig. 3:**
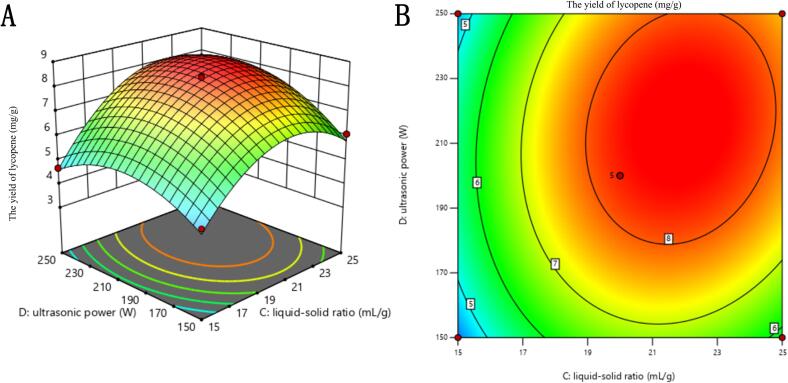
3D response surface graph (A) and contour plot (B) for the interactions of liquid–solid ratio and ultrasonic power on the lycopene yield.

To evaluate the interaction effects between two parameters, Both two-dimensional contour plots and three-dimensional response surface plots can be employed. Fig. 3 displayed the interaction effect (*X_3_X_4_*) between liquid–solid ratio (*X_3_*) and ultrasonic power (X_4_), with the other two parameters maintained at level zero in the figure presented. Since the *p*-values of other interacting factors were significantly greater than 0.1 (Table 3), they are not discussed in this section.

The effects of liquid–solid ratio and ultrasonic power on lycopene production were depicted in Fig. 3. Both the utilization of liquid–solid ratio (*X_3_*) and ultrasonic power (*X*_4_) contributed to an enhancement in lycopene yield. In Fig. 3A, it is evident that the lycopene yield exhibited a substantial increase with an escalating liquid–solid ratio; nevertheless, there was a marginal decline observed once a specific threshold was surpassed at higher ratios. Fig. 3B showed the variation trend of the lycopene yield was similar at different liquid–solid ratios as the ultrasonic power increases. Additionally, it can be observed that the impact of ultrasonic power on lycopene yield remained minimal at lower liquid–solid ratios but became more pronounced as the ratios increased. This phenomenon can be attributed to ultrasound cavitation, which is significantly influenced by both ultrasonic power and liquid–solid ratio. When the liquid–solid ratio is lower, even lower ultrasonic power levels are sufficient for cavitation to rupture plant cell walls; however, greater ultrasonic power levels are necessary when dealing with higher liquid–solid ratios.

### 3.4 ANN-GA optimization

**Fig. 4:**
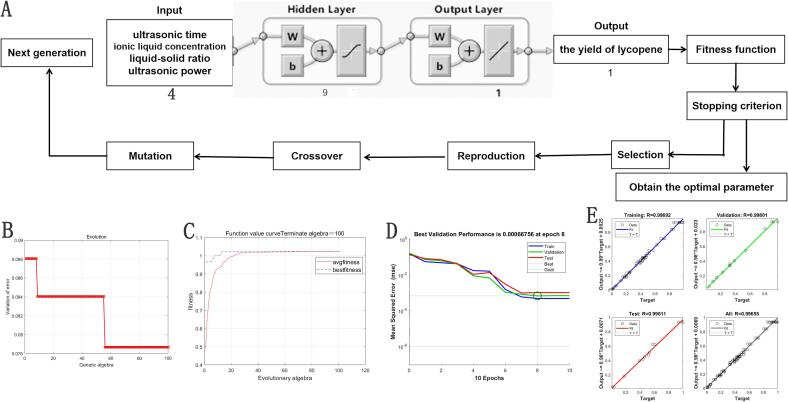
(A) optimal architecture of developing ANN-GA model, (B) error variation of the ANN-GA model with different generations, (C) fitness curve with evolutionary algebra, (D) network training curves with Epochs number for trained subsets, (E) regression coefficient of experimental data and ANN-GA model.

The link between the output variable and the four inputs was simulated using ANN-GA. The process of building the model involved gathering data, building the network, configuring it, initializing the weights and biases, training it, putting it to the test, and lastly verifying it [25]. The artificial neural network (ANN) was designed with three layers: an input layer with four neurons, a hidden layer with one neuron, and an output layer with one neuron. Therefore, ANN topology was 4–9–1 (Fig. 4A).

The change in the lycopene yield is gradually slower after 10 algebra, reaching the best fitness after 57 algebra. Subsequently, the average fitness curve was smooth, indicating that the lycopene yield had reached its maximum and remained essentially unchanged (Fig. 4B-C). Regarding the MSE parameter, the performance evaluation revealed that the network improved as the quantity of epochs increased while undergoing training, validation, and testing. With an MSE value of 6.6756E–04, the best validation performance was attained after 8 epochs (Fig. 4D). The results of the post-training analysis indicated a strong correlation between the expected (output) and the actual (target) data, with R values for training, validation, test, and all being 0.99692, 0.99801, 0.99611, and 0.99655, respectively (Fig. 4E).

### 3.5 Assessment of RSM and ANN-GA models' performance

**Fig. 5:**
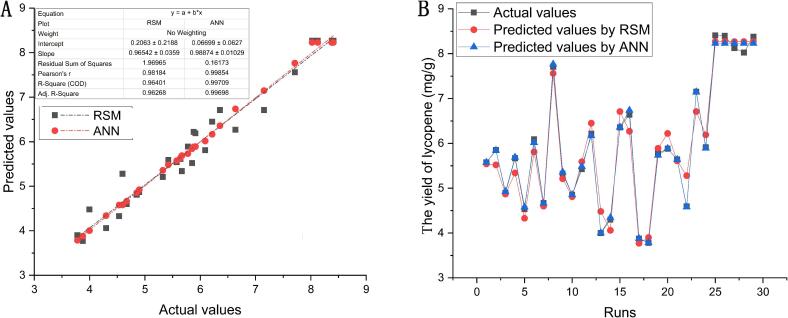
Diagnostic plots showing the adequacy of the developed RSM and ANN-GA models. (A) correlation of predicted values and experimental values by RSM, and ANN-GA models. (B) comparison plot between experimental BBD values and predicted values using RSM, and ANN-GA models.

A perfect match was found when the projected data produced by the two models was plotted against the experimental values (Fig. 5A), indicating that the models are extremely well constructed. However, it's crucial to note that, when comparing predictive capacity, ANN-GA prediction outperforms RSM with a greater R^2^ (0.99698 vs. 0.96226) and a smaller Residual Sum of Squares (0.16173 vs. 1.96965). Based on the R^2^ values, RSM and ANN-GA accounted for 96.226 % and 99.698 % of lycopene yield changes, respectively. Furthermore, compared with the predicted values by RSM, the values that the ANN-GA predicted were more in line with the actual values (Fig. 5B). Previous studies have shown that the ANN model is superior to the RSM model [26], [27].

### 3.6 Optimization of the process

Two distinct methods—RSM and ANN–GA—were considered to maximize lycopene extraction. As shown in Table 4, the optimal parameters suggested by RSM include an extraction time of 29.8 min, an ultrasonic power of 224.3 W, a liquid–solid ratio of 22.4 mL/g, and an IL concentration of 3.1 mol/L. On the other hand, ANN-GA running suggested an extraction time of 33.3 min, an ultrasonic power of 225.9 W, a liquid–solid ratio of 22.1 mL/g, and an IL concentration of 3.0 mol/L. Under this procedure, RSM and ANN-GA projected 8.44 and 8.57 mg/g yield, respectively. The two methods displayed disparate optimal settings. However, the verification of these analyses demonstrated a strong agreement between the estimated and verified lycopene recovery (8.52 mg/g and 8.66 mg/g, respectively, employing RSM and ANN–GA), demonstrating the effectiveness of both models in optimizing lycopene extraction and demonstrating the superiority of ANN-GA in this regard.

**Table 4:** Predicted and experimental values of the responses at optimum conditions.

	Optimum condition				**The yield of lycopene** (mg/g)		Relative error
	Extraction time (min)	IL concentration (mol/L)	liquid–solid ratio (mL/g)	ultrasonic power (W)	actual value	predicted	(%)
						value	
RSM	29.8	3.1	22.4	224.3	8.44	8.52	0.94
ANN	33.3	3	22.1	225.9	8.57	8.66	1.05

### 3.7 Effect of extraction cycles on extraction efficiency

**Fig. 6:**
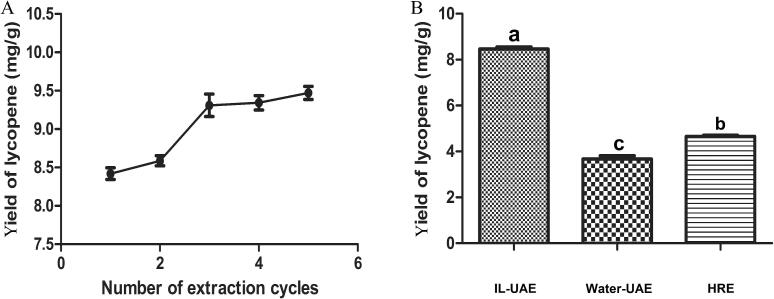
(A) effect of extraction cycles on the lycopene yield. (B) the lycopene yield comparison of the proposed IL-UAE, water UAE and HRE methods. Data are means ± SD, n = 3 for each bar. Different low case letters above columns indicate statistical differences at* p* < 0.05, same letters indicate insignificantly different (*p* > 0.05).

Even if the sample in the previously stated experiments was only extracted once, it is crucial to investigate the cycle of raw material extraction in order to improve the process's extraction efficiency. The extraction adjusted settings of ultrasonic power, liquid–solid ratio, IL concentration, and extraction time are 225 W, 22 mL/g, 3.0 mol/L, and 33 min, respectively, based on the findings of ANN-GA. Five extraction cycles of the same experiment were conducted to improve extraction cycles. As seen in Fig. 6A, three times' total extraction yield (9.35 ± 0.36 mg/g) was greater than 98.3 % if the five times' total extraction yield was considered as 100 % (9.51 ± 0.12 mg/g). Thus, ultrasonic extraction of lycopene needs to be carried out three times to optimize the utilization of the raw materials.

### 3.8 Comparing IL-UAE technique to the conventional methods

Water is always used as a co-solvent in many extraction processes since it is the most accessible and least costly solvent. Lycopene was extracted using pure water by UAE and 95 % ethanol by HRE to compare the extraction yield of IL-UAE. Fig. 6B showed the extraction yield of lycopene using pure water (3.67 ± 0.21 mg/g) was much lower than that using an IL aqueous solution (8.66 ± 0.29 mg/g), suggesting that IL performed a significant part in the extraction procedure. In comparison to the extraction efficiency of 95 % ethanol HRE (4.66 ± 0.34 mg/g), the recommended approach significantly shortened the overall extraction time while simultaneously improving extraction efficiency.

**Fig. 7:**
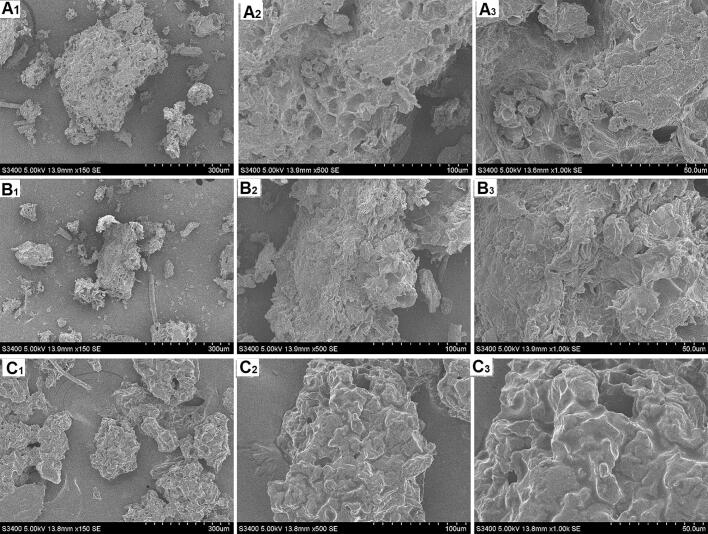
Microscopic morphology comparison by Scanning electron microscopy (SEM) of guava powder. (A: A_1_:×150; A_2_:×500; A_3_:×1000): sample residues obtained through hot reflux extraction (HRE), (B: B_1_:×100; B_2_:×500; B_3_:×1000): sample residues obtained through water UAE, (C: C_1_:×100; C_2_:×500; C_3_:×1000): sample residues obtained through IL-UAE.

SEM imaging allows for the observation of the microstructure to investigate the process mechanism. As a result, Fig. 7 depicts the morphological alterations of the guava samples subjected to various extraction techniques. The material treated with water UAE showed a somewhat wrinkled surface compared to the sample treated with 95 % ethanol HRE, which had a smooth and flat surface (Fig. 7A and B). There was evidence of destruction on the plant tissue's exterior with the IL UAE treatments (Fig. 7C). The water UAE picture (Fig. 7B2-B3) demonstrated ultrasonic wave's acoustic cavitation destroyed the cell wall and damaged the cell tissue, causing fractures and ruptures. In contrast, more cell tissue was destroyed in the IL UAE picture (Fig. 7C2-C3), illustrating the combined effects of IL and UAE approaches. These findings suggest that the suggested ionic liquid treatment in conjunction with UAE might be used to break down the plant tissue's microstructure, facilitating the solvent's easier entry into the sample and extraction of the desired chemicals. Consequently, IL-UAE is a quick and effective substitute for lycopene extraction. Based on the optimization findings in the laboratory, it has been suggested that ultrasonic technology may be considered on an industrial scale for UAE. As a result, certain optimization results of UAE of active chemicals from natural products have now been utilized in pilot size trials. Furthermore, the optimization results for IL-UAE of lycopene from guava will also provide valuable references for industrial-scale UAE of lycopene.

## 4 Conclusion

In this work, lycopene was extracted from the fruits of guava (*Psidium guajava* L.) using a newly created IL-UAE method. Based on the single-factor experiment, both RSM and ANN-GA were created, and their capacity to forecast the lycopene yield from guava was shown to be sufficiently reliable. However, in terms of performance, ANN-GA is more accurate. In contrast to RSM, ANN-GA produced the ideal process parameters, which included extraction time of 33 min, liquid–solid ratio of 22 mL/g, IL concentration of 3.0 mol/L, ultrasonic power of 225 W, and three extraction cycles, to achieve the highest lycopene yield (9.35 ± 0.36 mg/g). Furthermore, the inclusion of IL-UAE has demonstrated a good function in accomplishing full extraction as compared to conventional extraction procedures. In conclusion, the IL-UAE for lycopene is a technique with significant promise for market engineering applications and a high capacity for extracting important compounds from medicinal plants.

## CRediT authorship contribution statement

**Junping Wang:** Writing – original draft, Data curation, Conceptualization. **Hongyi Zhao:** Software, Methodology. **Xuexue Xue:** Validation, Data curation. **Yutong Han:** Methodology, Data curation. **Xin Wang:** Validation. **Zunlai Sheng:** Writing – review & editing, Writing – original draft, Funding acquisition, Conceptualization.

## Declaration of competing interest

The authors declare that they have no known competing financial interests or personal relationships that could have appeared to influence the work reported in this paper.

## References

[1] Liu H., Wei S., Shi L., Tan H. (2023). Preparation, structural characterization, and bioactivities of polysaccharides from Psidium guajava: A review. Food Chem..

[2] Pandey S.K., Joshua J.E., Bisen A. (2010). Influence of gamma-irradiation, growth retardants and coatings on the shelf life of winter guava fruits (Psidium guajava L.). J. Food Sci. Technol..

[3] Caseiro M., Ascenso A., Costa A., Creagh-Flynn J., Johnson M., Simoes S. (2020). Lycopene in human health. LWT-Food Sci. Technol..

[4] Lim J.Y., Wang X.-D. (2020). Mechanistic understanding of β-cryptoxanthin and lycopene in cancer prevention in animal models. Biochim. Biophys. Acta. Mol. Cell. Biol. Lipids..

[5] Petyaev I.M. (2016). Lycopene deficiency in ageing and cardiovascular disease. Oxid. Med. Cell. Longev..

[6] Leh H.E., Lee L.K. (2022). Lycopene: A potent antioxidant for the amelioration of type II diabetes mellitus. Molecules..

[7] Liu X., Lin X., Zhang S., Guo C., Li J., Mi Y., Zhang C. (2018). Lycopene ameliorates oxidative stress in the aging chicken ovary via activation of Nrf2/HO-1 pathway. Aging (albany NY)..

[8] Wawrzyniak D., Rolle K., Barciszewski J. (2023). Lycopene – the impact of supplementation on the skin aging process. Postepy Biochem..

[9] Wei M.Y., Giovannucci E.L. (2012). Lycopene, tomato products, and prostate cancer incidence: a review and reassessment in the PSA screening era. J. Oncol..

[10] Cui L., Xu F., Wu K., Li L., Qiao T., Li Z., Chen T., Sun C. (2020). Anticancer effects and possible mechanisms of lycopene intervention on N-methylbenzylnitrosamine induced esophageal cancer in F344 rats based on PPARγ^1^. Eur. J. Pharmacol..

[11] Gharib A., Faezizadeh Z. (2014). *In vitro* anti-telomerase activity of novel lycopene-loaded nanospheres in the human leukemia cell line K562. Pharmacogn. Mag..

[12] Goula A.M., Adamopoulos K.G., Chatzitakis P.C., Nikas V.A. (2006). Prediction of lycopene degradation during a drying process of tomato pulp. J. Food Eng..

[13] Shen L., Pang S., Zhong M., Sun Y., Qayum A., Liu Y., Rashid A., Xu B., Liang Q., Ma H., Ren X. (2023). A comprehensive review of ultrasonic assisted extraction (UAE) for bioactive components: Principles, advantages, equipment, and combined technologies. Ultrason. Sonochem..

[14] Chmelova D., Skulcova D., Legerska B., Hornik M., Ondrejovic M. (2020). Ultrasonic-assisted extraction of polyphenols and antioxidants from Picea abies bark. J. Biotechnol..

[15] Belwal T., Ezzat S.M., Rastrelli L., Bhatt I.D., Daglia M., Baldi A., Devkota H.P., Orhan I.E., Patra J.K., Das G., Anandharamakrishnan C., Gomez-Gomez L., Nabavi S.F., Nabavi S.M., Atanasov A.G. (2018). A critical analysis of extraction techniques used for botanicals: Trends, priorities, industrial uses and optimization strategies, Trac-trend. Anal. Chem..

[16] Qiu J., Shi M., Li S., Ying Q., Zhang X., Mao X., Shi S., Wu S. (2023). Artificial neural network model- and response surface methodology-based optimization of Atractylodis Macrocephalae Rhizoma polysaccharide extraction, kinetic modelling and structural characterization. Ultrason. Sonochem..

[17] Jovanović M., Mudrić J., Drinić Z., Matejić J., Kitić D., Bigović D., Šavikin K. (2022). Optimization of ultrasound-assisted extraction of bitter compounds and polyphenols from willow gentian underground parts. Sep. Purif. Technol..

[18] Hou K., Bao M., Wang L., Zhang H., Yang L., Zhao H., Wang Z. (2019). Aqueous enzymatic pretreatment ionic liquid-lithium salt based microwave-assisted extraction of essential oil and procyanidins from pinecones of *Pinus koraiensis*. J. Cleaner Prod..

[19] Peng X., Sui X., Li J., Liu T., Yang L. (2021). Development of a novel functionality for a highly efficient imidazole-based ionic liquid non-aqueous solvent system for the complete extraction of target alkaloids from Phellodendron amurense Rupr. under ultrasound-assisted conditions. Ind. Crops Prod..

[20] Cerqueira S.C.A., Santos L.M., Gois A.R.S., Soares C.M.F., Neto B.A.D., Freitas L.S. (2023). Use of Ionic Liquid-Based Ultrasound Assisted Liquid-Liquid Extraction of Phenols from Aqueous Fractions of Seed Bio-Oil. J. Braz. Chem. Soc..

[21] Qin H., Zhou G., Peng G., Li J., Chen J. (2015). Application of Ionic Liquid-Based Ultrasound-Assisted Extraction of Five Phenolic Compounds from Fig (Ficus carica L.) for HPLC-UV. Food Anal. Methods..

[22] Mk A., Bk S. (2020). Isolation and Quantification of Lycopene and Determination of Β-Carotene and Total Phenolic Contents from Tomato (Lycopersicum Esculentum) by using Various Methods. Int. J. Food Sci. Nutr. Diet..

[23] Wang W., Li Q., Liu Y., Chen B. (2015). Ionic liquid-aqueous solution ultrasonic-assisted extraction of three kinds of alkaloids from Phellodendron amurense Rupr and optimize conditions use response surface. Ultrason. Sonochem..

[24] Yang L., Wang H., Zu Y.-G., Zhao C., Zhang L., Chen X., Zhang Z. (2011). Ultrasound-assisted extraction of the three terpenoid indole alkaloids vindoline, catharanthine and vinblastine from *Catharanthus roseus* using ionic liquid aqueous solutions. Chem. Eng. J..

[25] Khajeh M., Moghaddam M.G., Shakeri M. (2012). Application of artificial neural network in predicting the extraction yield of essential oils of Diplotaenia cachrydifolia by supercritical fluid extraction. J. Supercrit. Fluids..

[26] Phong N.V., Gao D., Kim J.A., Yang S.Y. (2023). Optimization of ultrasonic-assisted extraction of alpha-glucosidase inhibitors from dryopteris crassirhizoma using artificial neural network and response surface methodology. Metabolites..

[27] Aklilu E.G. (2021). Modeling and optimization of pectin extraction from banana peel using artificial neural networks (ANNs) and response surface methodology (RSM). J. Food Meas. Charact..

